# The Metropolitan Workhouse Infirmaries

**Published:** 1890-08-23

**Authors:** 


### The Practitioner's Mirror and Retrospect.

*[The Editor will be glad to receive offers of co-operation and contributions from members of the profession. There is no doubt that much valuable material full of interest and instruction is at present lost to science. This is largely so in the case of (1) the younger and less-known members of the hospital staff (2) those connected with cottage hospitals, and (3) the great body of medical practitioners not attached to any institution. The co-operation of all is cordially invited to enable the Editor to make* The Hospital *of the utmost possible use and value to the profession. Specialists willing to review books on their special subjects are invited to communicate this fact at once. All letters should be addressed to* The Editor, The Lodge, Porchester Square, London, W.]

### THE METROPOLITAN WORKHOUSE INFIRMARIES.

### Resolution of the Paddington Board of Guardians.

The adoption by the Paddington Board of Guardians of the following resolution, proposed by Dr. Felce, at their meeting on February 10th, 1890, will give those interested in the question of medical relief ample material for reflection:—"That Professor J. Hutchinson and Dr. Broadbent be invited to examine the cases in the Infirmary in consultation with the Medical Superintendent, and deliver lectures at the Infirmary in connection with the London Post Graduate course now in process of formation. It being understood that such a procedure is not attended with any additional cost to the ratepayers, and does not interfere with the existing management and discipline of the institution." This action on the part of the Paddington Board is only one of the evidences of a general movement, and a change in public opinion, which will, it is hoped, gradually result in a considerable improvement being made in the administration of medical relief to a large section of the sick poor of the Metropolis.

### Medical Institutions of London.

There are five great classes of medical institutions belonging to the Metropolis, into which a person who is ill, either in mind or body, and unable to pay for his food and medical attendance at home, can be received.

1. The *Lunatic Asylums* of Banstead, Cane Hill, Colney Hatch, and Hanwell, formerly controlled by the Justices, now by the County Council, and containing 7,279 beds.*
2. The *Imbecile Asylums* of Darenth, Caterham, and Leavesden, which receive idiots and harmless lunatics, and are controlled by the Metropolitan Asylums Board, containing 6,194 beds.†
3. The *Fever Hospitals and Ships*, nine in number (excluding the London Fever Hospital), for the reception of cases of small-pox, scarlet fever, diphtheria, &c., also governed by the Metropolitan Asylums' Board, containing 2,800 beds.‡
4. The *Special and General Hospitals*, 92 in number, for the reception of accidents and all kinds of illness not received at the first three (excepting the London Fever Hospital, which takes in fever cases only), supported by voluntary contributions or endowments, and each governed by its own administrative, containing 9,173 beds (of which about three-quarters are surgical).§
5. The *Poor Law or Workhouse Infirmaries*, 24 in number, also for the reception of general diseases; supported partly by the poor rates of their respective parishes, and partly out of the Metropolitan Common Poor Fund; governed by the Guardians of the Poor of their respective parishes, and containing a total number of 12,170 beds (at least three-quarters of which are medical).‖

*Hanwell 1,894, Colney Hatch 2,259, Banstead 2,002, Cane Hill 1,124.

†Darenth 2,144, Caterham 2,050, Leavesden 2,000.

‡Eastern Fever Hospital 542, North-Western Hospital 435, South-Western Hospital 402, Western Hospital 262, South-Eastern Hospital 393, Northern Hospital 416, and three hospital ships 350.

§Vide Medical Directory.

‖Mile End Infirmary 469, St. George's-in-the-East 416, Marylebone 744, Lambeth 622, Chelsea 386, Central London Sick Asylum 264, Kensington 620, St. Saviour's Infirmary 786, Fulham 486, Hackney 437, St. Mary's, Islington, 540, City of London Union 645, Greenwich 247, Poplar and Stepney Sick Asylum 586, Holborn Union 625, Shoreditch 472, Camberwell 331, St. Pancras 523, Wandsworth 618, St. Olave's 388, Woolwich 216, St. George's Union (Hanover Square) 776, Whitechapel 689, Paddington 284. The only poor law parish not possessing an infirmary at the present time is Bethnal Green.

In view of the enquiry by the Committee of the House of Lords now sitting to consider the question of medical relief in the Metropolis these facts are particularly interesting. It is only since October, 1886, that the authorities of the Poor Law infirmaries have been enabled, by an amending order of the Local Government Board, issued at the instigation of the Paddington Board of Guardians, to admit cases of accident or other severe illness without the order of the relieving officer of the parish. Consequently it is only since this date that the State has provided for street accidents. All other forms of illness were provided for by Classes 1, 2, 3, and 5, which, it will be seen, are State institutions, but accidents, cases of poisoning, drowning, and other emergencies were left for private charity to meet by means of Class 4. This was a most important concession, and it did away with one of the four fundamental differences between a hospital and an infirmary, the other three distinctions being that the hospitals (1) are supported by endowments or private charity instead of poor rates; (2) have an adequate medical staff; and (3) admit students of medicine to their wards. It will be seen that Classes 1, 2, and 3 are for the reception of special kinds of disease which are very largely drafted off from, or filtered through the Hospitals (Class 4) and Infirmaries (Class 5), to which, as a matter of fact, the first three are annexes.

### Origin of Infirmaries.

It is with the Infirmaries alone that we are now concerned, and it will be interesting to turn for a moment to their origin and evolution. Prior to the year 1865 the condition of the London workhouses was something deplorable. The sick and the well were all indiscriminately huddled together. The chaos and abuses which existed in some are not overdrawn in the works of Charles Dickens. There was, however, one bright spot in the darkness—Marylebone Workhouse, where there existed an energetic medical officer (albeit non-resident), who was aided by several visiting physicians and surgeons, several of whom subsequently achieved eminence.

About the year 1865 an agitation was started, chiefly owing to the revelations of the "*Lancet* Commission," of Miss Louisa Twining and other private individuals, which resulted in the passing of the Metropolitan Poor Law Act of 1867 (Gathorne Hardy's Act). Amongst other reforms, this Act empowered guardians to build separate institutions (Infirmaries), distinct from the workhouse of the parish, for the reception of the sick indoor poor. Separate "regulations" were issued by the Local Government Board for the government of these new buildings, in which two features always stood out prominently. First, that the chief administrative authority should rest in the hands of a medical man, *who should be resident*; and, secondly, that the nursing should be performed by a properly trained staff of paid nurses, in place of the pauper system formerly in vogue. The medical superintendent was to have one resident assistant, and one or other of them was always obliged to be on the premises in case of need. This Act of 1867 expressly gave permission, in Section 29, to use these new buildings for the purposes of medical instruction and for the training of nurses; but this permission was unfortunately repealed, for reasons which have never been apparent, in Section 20 of an amending Act passed two years later.

One by one have these buildings sprung up, sometimes, as at Kensington, adjacent to the parish workhouse, sometimes, as at Marylebone, a little way out of London. So silently and steadily has their growth taken place that the fact of their existence has scarcely come to be realised; and it is by no means generally known that the twenty-four metropolitan Infirmaries (Bethnal Green Infirmary is not yet completed), contain an aggregate of 12,170 beds as against 9,173 in all the special and general hospitals of the Metropolis put together. The Infirmaries vary considerably in size, from 216 beds at Woolwich to 786 at St. Saviour's Union. But this important fact remains that, excluding the lunatic and imbecile asylums and fever hospitals, *more than half of the indoor sick poor of this great city are lodged in the Workhouse Infirmaries.* The hospitals, especially the eleven to which medical schools are attached, largely contain surgical cases, and the infirmaries chiefly medical cases.

These latter are, as we have said, almost unknown to the general public, who are shut out from them. There are no students to criticise the doctors' methods of diagnosis and treatment, and keep them up to their work; no clinical clerks and dressers to help them to investigate and treat the patients; no consultants to advise in cases of difficulty. These great storehouses of disease are closed to the outer world. Even the post of medical officer has, until late years, been looked upon as a comfortable appointment upon which to retire from active practice, with a small but certain income attached, which none of the distinguished among the younger men would compete for on account of the manner in which the doctor was usually treated by his board, and the indignities he had to suffer when he ventured to "advise."

The medical staff of these institutions has consisted until quite recently of only two medical men, both resident, one of whom is charged also with the chief administrative control, and, in some instances, the other is the responsible if not the actual dispenser. These gentlemen, therefore, have under their care an average number of 253 beds each in addition to their other duties, and the question may fairly be asked, how is it possible that they can do their duty towards the patients placed under their care?

### Class of Case.

It is true that the class of case in an infirmary differs somewhat from that in a hospital, but on the other hand, all the cases in the former are not chronic and incurable, far from it, as the records of our various learned societies show. It is also true that the number of surgical cases (which take more time than the medical) is considerably smaller than in hospital work. But this difference is more than compensated for by the large staff of dressers and clinical clerks attached to every big hospital. Compare, for instance, the medical staff at St. Mary's Hospital (280 beds), which consists of four visiting physicians, four visiting surgeons, eight resident house surgeons and house physicians (not counting the out-patient visiting staff, or the senior students who act as clerks and dressers), with that of the Central London Sick Asylum (264 beds) which consists of two residents *only*, neither of whom can devote their undivided attention to medical work. It is clear that either the staff of the one is unnecessarily great, or that of the other ridiculously inadequate. It is, moreover, an undoubted fact, on which stress was laid by Dr. Bridges in his evidence before the Lords' Committee on Poor Law Relief in 1888, that a very large number of obscure forms of paralysis and other chronic ailments get into these infirmaries, which are more or less curable if they are only submitted to thorough investigation and systematic treatment, such as cannot be afforded by the existing inadequate staff, *for they require more attention than even a difficult surgical case.*

### Consequences.

The unhappy result of such a state of things is threefold. To the patient who might be cured, there is loss of health and perhaps of life; to the ratepayer, an unnecessary expense in the maintenance of persons who might become useful members of the community, instead of remaining a burden to the rates; and to medical science a loss of valuable clinical material for experience (*not* experiment) and observation. Hence it is clear from every point of view that anything which tends to promote the more complete investigation and thorough treatment of the inmates of the infirmaries is a most desirable achievement; and an improvement in the medical staff, by accomplishing this end, would be a benefit, not only to the patient, but also to the ratepayer, who is bound to maintain him, and very likely his family, during his sickness; and lastly, to medical science, which is thus afforded a fresh field for usefulness and experience.

### Remedies.

How this improvement of staff may best be effected is therefore a question which deserves earnest consideration. It has been vaguely suggested to throw open these institutions to the medical world; to let in the light; to amalgamate them with or turn them into medical schools. But unfortunately, as we have pointed out, there are serious legal difficulties in the way of this. It has been suggested to appoint consultants, who should visit and advise, or perhaps take medical charge of the patients. Then there is the plan of increasing the resident staff. This reform is very greatly needed, and should certainly come first, whatever else follows. There are no legal difficulties in the way; and if only carried out with sufficient boldness and liberality it would meet all the indications. Another plan, about the legality of which some difference of opinion exists, is the appointment of a limited number of non-resident dressers and clinical clerks. For our own part we regard this as the most practical and feasible suggestion yet made.

In 1886 the Paddington Guardians adopted a scheme for the appointment of a resident clinical assistant, a qualified medical man, to hold office for six months, receive board and an honorarium of twelve guineas in return for his services. There was, unfortunately, some delay in the execution of this arrangement, which, by providing a third resident medical man, would promote the elucidation of cases, and would offer great advantages to a young man fresh from the schools where surgical and obscure medical cases are almost the only ones seen. We believe that this scheme is now in force and that it works well.

A very similar scheme has been in operation at the Marylebone Infirmary for nearly two years, and a not altogether dissimilar one has been in working at the St. Pancras Infirmary for the same time.

### Good Results.

If the other Infirmaries would follow the lead, it might safely be prophesied that great good would be done for the sick poor of London, and in the end true economy, since it is based on efficiency, would result. And what an advantage to the newly-qualified practitioner would be these twenty-four appointments, which would be available every six months, wherein he could gain experience, under proper supervision, of a large and varied class of ailments that are not sufficiently, if at all, seen in hospital wards (such as bronchitis, emphysema, chronic rheumatism, chronic gout, and nerve diseases), yet which really make up a large proportion of the cases he sees in ordinary private practice. And what an advantage to the medical science of the future would be the opening up of this vast unexplored field of so-called incurable ailments amongst patients who have been the round of the hospitals, and who gravitate inevitably to the infirmary, there to spend their concluding days.

The latest proposal of the Paddington Board of Guardians quoted at the commencement of this article is undoubtedly a move in the right direction, and ought to throw much light on the cases and help the resident staff in their medical work without relieving them of the responsibility, which should always attach to them, of the care and proper treatment of the patients. The Post Graduate Classes have been formed to supply a want felt by gentlemen who have finished their pupilage and have entered the profession, perhaps some years before, but who have a desire to learn something more without joining a school of medicine and mixing with the beginners. This proposal, while it will give these gentlemen an opportunity of seeing the cases, will also tend to a more thorough investigation of the patients, the detection of malingerers, and a more efficient system of treatment; and withal under due restriction will not affect the otherwise excellent discipline and management of the Infirmary.

We shall watch the working of the new departure with the greatest interest. That the resident staff of these institutions is sadly in need of increase is abundantly evident. We congratulate the ratepayers of Paddington in possessing such an enlightened and able Board of Guardians who are evidently aware of the true interests, both of those whom they represent and the destitute sick whose interests they are elected to guard.

